# Haemolytic differential identification of *Arcanobacterium haemolyticum* isolated from a patient with diabetic foot ulcers

**DOI:** 10.1099/jmmcr.0.005016

**Published:** 2016-02-12

**Authors:** Hyesook Kang, Gyunam Park, Hyeran Kim, Kyungsoo Chang

**Affiliations:** Department of Clinical Laboratory Science, College of Health Sciences, Catholic University of Pusan, Busan 609-757, Korea

**Keywords:** *Arcanobacterium haemolyticum*, CAMP inhibition test, diabetic foot ulcers, haemolytic differential method, reverse CAMP test

## Abstract

**Introduction:**

*Arcanobacterium haemolyticum* (formerly known as *Corynebacterium haemolyticum*) is the causative agent of sore throat and also causes skin and soft tissue infections in diabetes patients. *A. haemolyticum* is a Gram-positive, catalase-negative, β-haemolytic bacillus. *A. haemolyticum* poses a diagnostic challenge in the hospital laboratory because most coryneform bacilli are considered as normal flora or contaminants, and it is therefore difficult to differentiate from β-haemolytic streptococci by colony characteristics.

**Case presentation:**

*A. haemolyticum* was isolated from a diabetic patient with foot ulcers and the isolate was identified by using a VITEK-2 system, CAMP inhibition test, reverse CAMP test and a 23S rRNA gene sequence analysis. The isolated *A. haemolyticum* inhibited haemolysis of *Staphylococcus aureus* in the CAMP test and enhanced haemolysis of *Streptococcus agalactiae* in the reverse CAMP test. The diabetic patient was treated with teicoplanin and imipenem, and the ulcers healed within 2 weeks.

**Conclusion:**

The present study suggests that a haemolytic differential method using the CAMP inhibition and reverse CAMP tests can be useful for differentiating *A. haemolyticum* from β-haemolytic streptococci.

## Introduction

*Arcanobacterium haemolyticum* (formerly known as *Corynebacterium haemolyticum*) is the causative agent of skin infections and sore throat such as exudative pharyngitis and tonsillitis. It was first described by MacLean in 1946 (MacLean *et al.*, 1946; Collins *et al.*, 1982). *A. haemolyticum* is known to be part of the normal flora on the throat and skin, and mainly causes sore throat in young people. Occasionally, it causes skin and soft tissue infections, and in rare cases it can cause osteomyelitis, pneumonia, endocarditis and septicaemia (Waller *et al.*, 1991; Mackenzie *et al.*, 1995). *A. haemolyticum* is a β-haemolytic, Gram-positive, rod-shaped and catalase-negative bacillus, and can be characterized by its ability to inhibit β-haemolysis in a CAMP test. In the present study, we report a case of *A. haemolyticum* isolated from a patient with diabetic foot ulcers.

## Case report

**Fig. 1.**
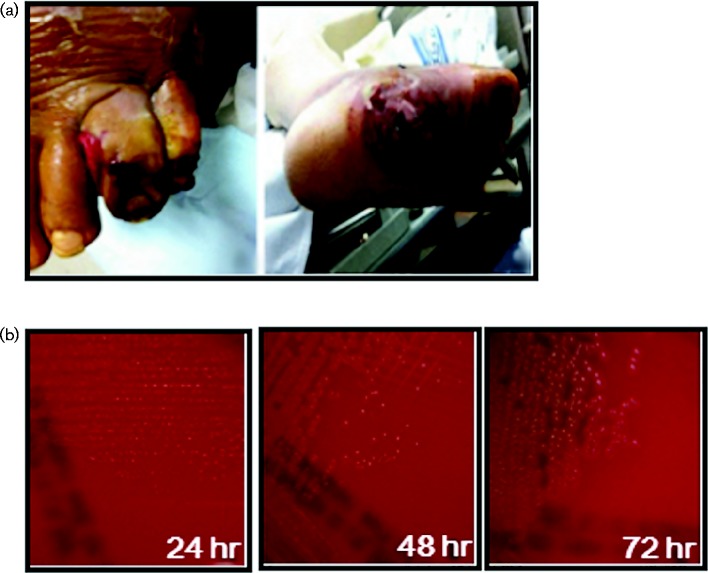
Diabetic ulcers and colony changes. (a) Diabetic ulcers on the feet of the patient, who has been suffering from chronic diabetes. (b) Colony changes of *A. haemolyticum* on an SBA plate over time.

A 42-year-old male patient suffered from severe oedema, fever and pain from deep wounds on both feet that were not the result of trauma at the time of hospitalization. The deep wound infection on his left foot also had a foul odour (Fig. 1a, right panel). The patient had been diagnosed previously as having diabetes with high blood pressure and had been treated with hypoglycaemic and antihypertensive enhancers orally, as well as insulin intravenously. At the time of hospitalization, the patient's vital signs were a body temperature of 38.7 °C, blood pressure of 110/70 mmHg, a pulse of 70 beats min^− 1^ and a respiratory rate of 20 beats min^− 1^. The patient's body temperature rose steadily to a peak of 39.9 °C. The number of leukocytes and the level of C-reactive protein in peripheral blood were determined to be 19 000 mm^− 3^ and 202.42 mg l^− 1^, respectively. During hospitalization, samples from his foot lesions were collected nine times for bacterial culture and blood samples were collected for blood cultures. Blood cultures were conducted a total of four times with three sets each time. The patient was administered intravenously with ciprofloxacin following hospitalization in accordance with empirical therapy procedures.

## Diagnosis

The samples from the foot lesions were cultured on 5 % sheep blood agar (SBA) and a MacConkey agar plate under conditions of 5 % CO_2_ for 24 h. Very small grey colonies without observable β-haemolysis were found on the SBA plate (Fig. 1b, left panel), but no colonies were found on the MacConkey agar plate (data not shown). The isolates from the colonies were identified as Gram-positive rods by Gram staining and a catalase reaction was negative (data not shown). The colonies became more distinct and displayed a small degree of β-haemolysis after 48 h of culture (Fig. 1b, middle panel). Small smooth grey colonies with distinct β-haemolysis were observed after 72 h of culture (Fig. 1b, right panel). The isolate was identified as *A. haemolyticum* (99 %) by the VITEK-2 automated microbiology system (BioMérieux) (Table 1). Blood samples were incubated four times in the BACTEC 9240 blood culture system (BD) for 5 days, but all blood cultures were negative (data not shown).

**Table 1.** Comparison of biochemical properties and haemolysis activities between the *A. haemolyticum* isolate and reference strain ATCC 9345 by VITEK-2 and CAMP tests

Biochemical reaction	*A. haemolyticum*ATCC 9345	*A. haemolyticum*clinical isolate
Catalase	−	−
Urease	−	−
Glucose	+	V
Maltose	−	−
Sucrose	−	−
Xylose	−	−
Aesculin hydrolysis	−	−
CAMP reaction	I	I
Reverse CAMP	+	+

+, Positive; − , negative; V, variable; I, inhibition.

A CAMP inhibition test was then performed with the isolated *A. haemolyticum* in order to confirm its identity. *Staphylococcus aureus* ATCC 25923 was streaked in a straight line across the centre of the SBA plate (Fig. 1a, indicated as ‘a’). The isolated *A. haemolyticum* was streaked in a straight line perpendicular to the *Staphylococcus aureus* streak (Fig. 1a, indicated as ‘b’) in the lower left. *Streptococcus agalactiae* ATCC 13813 was streaked (Fig. 1a, indicated as ‘c’) similarly in the upper right as a positive control for haemolysis.

**Fig. 2.**
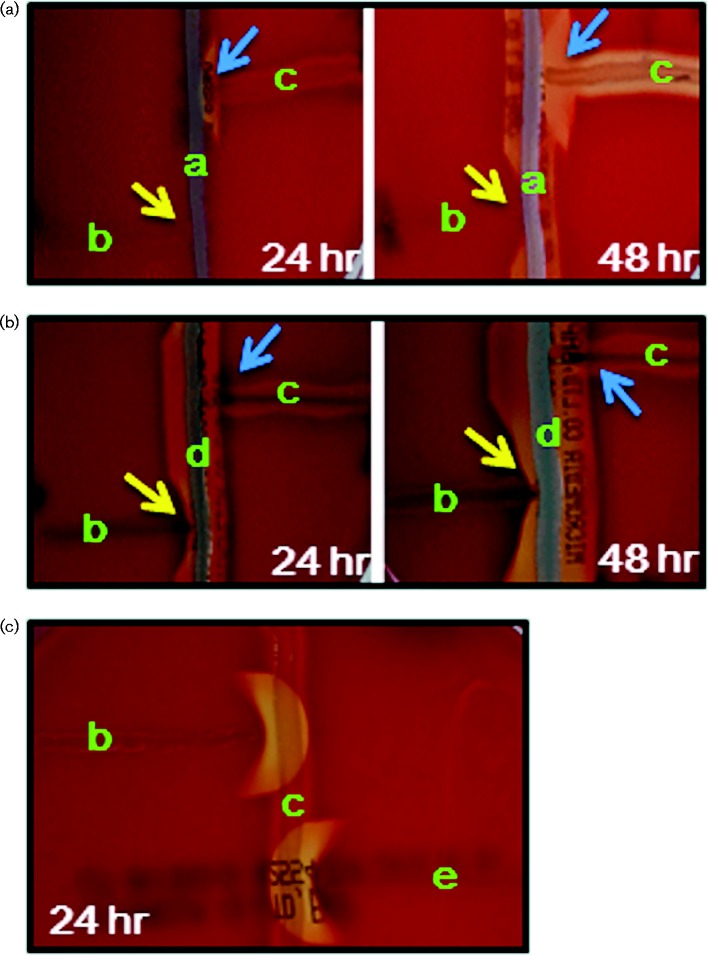
CAMP inhibition and reverse CAMP reaction of *A. haemolyticum*. (a, b) *A. haemolyticum* isolate in the lower left and *Streptococcus agalactiae* in the upper right were streaked perpendicularly to vertically streaked *Staphylococcus aureus* (ATCC 25923) (a) and *Staphylococcus aureus* (ATCC 29213) (b) on an SBA plate. (c) Reverse CAMP reaction of the isolated *A. haemolyticum* in the upper left and *A. haemolyticum* (ATCC 9345) in the lower right against *Streptococcus agalactiae* (ATCC 13813), which developed crescent-shaped haemolysis. ‘a’, *Staphylococcus aureus* (ATCC 25923); ‘b’, *A. haemolyticum* isolated from the patient; ‘c’, *Streptococcus agalactiae* (ATCC 13813); ‘d’, *Staphylococcus aureus* (ATCC 29213); ‘e’, *A. haemolyticum* (ATCC 9345).

The characteristic haemolysis inhibition by *A. haemolyticum* was not observed after 24 h of culture (Fig. 2a, yellow arrow in left panel) but became apparent after 48 h (Fig. 2a, yellow arrow in right panel). However, the arrow shape of haemolysis by *Streptococcus agalactiae* (ATCC 13813) occurred against *Staphylococcus aureus* ATCC 25923 after 24 h (Fig. 2a, blue arrow in left panel) and became more apparent after 48 h (Fig. 2a, blue arrow in right panel). In addition, we used haemolytic-sensitive strain *Staphylococcus aureus* ATCC 29213 in Fig. 2(b). The isolated *A. haemolyticum* haemolysis inhibition using *Staphylococcus aureus* ATCC 29213 was apparent within 24 h (Fig. 2b, yellow arrow in left panel) and increased after 48 h (Fig. 2b, yellow arrow in right panel). In contrast to the result in Fig. 2(a), haemolysis by *Streptococcus agalactiae* was not observed (Fig. 2b, blue arrow in both panels) due to enhanced haemolysis in *Staphylococcus aureus* ATCC 29213.

In the reverse CAMP test, the isolate *A. haemolyticum* was streaked in the upper left (Fig. 2c) perpendicular to the *Streptococcus agalactiae* streak. The waxing crescent-shaped haemolysis on the contact surface was observed clearly after 24 h. The control *A. haemolyticum* ATCC 9345 was streaked in the lower right (Fig. 2c), perpendicular to the *Streptococcus agalactiae* streak. Similar to the isolate *A. haemolyticum*, the waning crescent-shaped haemolysis on the contact surface was observed clearly after 24 h. Finally, the isolated *A. haemolyticum* was confirmed by genotyping using 23S rRNA gene PCR and sequencing. The sense and antisense primers were 5′-TAACGGTCCTAAGGTACCGA-3′ and 5′-GATAGGGACCGAACTGTCTC-3′, respectively. The sequencing result revealed that the isolated *A. haemolyticum* shared 99.9 % identity with *A. haemolyticum* DSM 20595 (data not shown).

## Treatment

The patient was administered intravenously with ciprofloxacin following hospitalization in accordance with empirical therapy procedures, but recovery was not observed in the early stages of treatment. There were no official guidelines for imipenem treatment available for the isolated *A. haemolyticum* by the Clinical and Laboratory Standards Institute although this pathogen was identified from the patient. Therefore, we used a similar *Corynebacterium* sp. as a reference. We performed an antibiotic susceptibility test with the isolated *A. haemolyticum* to teicoplanin and imipenem (in the carbapenem class of antibiotics) using an E-test. The MICs of teicoplanin and imipenem were 0.47 and 0.016 μg ml^− 1^, respectively. The patient was administered intravenously with imipenem twice per day and teicoplanin once every 2 days according to the results of the antibiotic susceptibility test. The vital signs of the patient returned to normal, and additional bacterial growth was not detected after 2 weeks of treatment with antibiotics.

## Discussion

Although *A. haemolyticum* isolated from respiratory samples typically produces rough colonies without β-haemolysis, the isolate from the wound skin infection produced smooth colonies with haemolysis (Carlson *et al.*, 1994). As expected, the isolated *A. haemolyticum* was identified as a β-haemolytic coryneform bacillus. Because coryneform bacilli are considered part of the normal flora or a contaminant, they are more difficult to identify (Meyer & Reboli, 2005).

The morphological characteristics of *A. haemolyticum* should be distinguished from the irregular shape of *Corynebacterium* spp. as Gram-positive rods and from *Streptococcus* spp. The differences between *A. haemolyticum* and *Corynebacterium* spp. are their colony shape on an agar plate and catalase positivity. However, comparisons between *A. haemolyticum* and catalase-negative streptococci, particularly β-haemolytic *Streptococcus* spp., do not yield distinct differences in colony shape when grown on 5 % SBA plate or in the catalase test. Thus, the methods in previous studies cannot differentiate between *A. haemolyticum* and catalase-negative *Streptococcus*.

In order to correctly identify *A. haemolyticum*, a CAMP inhibition test and reverse CAMP test should be used in conjunction with commercially available biochemical property test kits, colony size, morphology and haemolysis according to culture time. In this case, the haemolysis pattern of the *A. haemolyticum* isolate changed with culture time (from unclear β-haemolysis to weak β-haemolysis to distinct β-haemolysis after 24, 48 and 72 h of culture, respectively). *A. haemolyticum* inhibited the β-haemolysis of *Staphylococcus aureus* (ATCC 25923 and 29213). In addition, the CAMP inhibition and reverse CAMP tests using *Streptococcus agalactiae* were positive. Several previous studies have confused CAMP inhibition (β-haemolysis inhibition) with reverse CAMP (β-haemolysis enhancement) (Kim *et al.*, 2004; Bae *et al.*, 2010).

This report sought to redefine the role of the CAMP inhibition and reverse CAMP tests in identifying and differentiating *A. haemolyticum* from β-haemolytic streptococci using *Staphylococcus aureus* and *Streptococcus agalactiae*. In the CAMP inhibition test, *Staphylococcus aureus* ATCC 29213 caused more haemolysis inhibition than *Staphylococcus aureus* ATCC 25923 (Fig. 2a, b). The reverse CAMP test with the isolated *A. haemolyticum* from the diabetic patient exhibited a waxing crescent-shaped haemolysis, and the reference *A. haemolyticum* ATCC 9345 exhibited a waning crescent shape haemolysis (Fig. 2c).

In conclusion, this report suggests that haemolytic differential identification using CAMP and reverse CAMP tests might be a more effective method to differentiate *A. haemolyticum* from other Gram-positive coryneform bacillus and β-haemolytic streptococci.
